# Nanocarriers for Protein Delivery to the Cytosol: Assessing the Endosomal Escape of Poly(Lactide-co-Glycolide)-Poly(Ethylene Imine) Nanoparticles

**DOI:** 10.3390/nano9040652

**Published:** 2019-04-23

**Authors:** Marianna Galliani, Chiara Tremolanti, Giovanni Signore

**Affiliations:** 1 Center of Nanotechnology Innovation @NEST, Istituto Italiano di Tecnologia, 56127 Pisa, Italy; 2 NEST, Scuola Normale Superiore, 56127 Pisa, Italy; 3 Department of Pharmacy, University of Pisa, 56126 Pisa, Italy; chiara.tremolanti@phd.unipi.it; 4 Istituto di Fisiologia Clinica, National Research Council, 56124 Pisa, Italy; 5 Fondazione Pisana per la Scienza ONLUS, 56121 Pisa, Italy

**Keywords:** PLGA nanoparticles, endosomal escape, translocation, cytosol delivery, protein delivery

## Abstract

Therapeutic proteins and enzymes are a group of interesting candidates for the treatment of numerous diseases, but they often require a carrier to avoid degradation and rapid clearance in vivo. To this end, organic nanoparticles (NPs) represent an excellent choice due to their biocompatibility, and cross-linked enzyme aggregates (CLEAs)-loaded poly (lactide-co-glycolide) (PLGA) NPs have recently attracted attention as versatile tools for targeted enzyme delivery. However, PLGA NPs are taken up by cells via endocytosis and are typically trafficked into lysosomes, while many therapeutic proteins and enzymes should reach the cellular cytosol to perform their activity. Here, we designed a CLEAs-based system implemented with a cationic endosomal escape agent (poly(ethylene imine), PEI) to extend the use of CLEA NPs also to cytosolic enzymes. We demonstrated that our system can deliver protein payloads at cytoplasm level by two different mechanisms: Endosomal escape and direct translocation. Finally, we applied this system to the cytoplasmic delivery of a therapeutically relevant enzyme (superoxide dismutase, SOD) in vitro.

## 1. Introduction

Protein delivery has witnessed growing interest in the last decades, since proteins and enzymes can find application in the treatment of numerous diseases such as cancer, diabetes, vascular dysfunctions and metabolic disorders [1]. However, the simple systemic administration of therapeutic proteins to patients is rarely successful. In fact, these molecules are prone to degradation in the bloodstream and often fail in reaching their pharmacologic target in the organism. For this reason, efforts have been made to develop micro- and nanosized carriers made of various materials [2,3,4,5,6] able to safely deliver these delicate therapeutics to their site of action [7]. In this context, polymeric nanoparticles (NPs) [8,9], in particular those composed of biodegradable polymers like poly (lactide-co-glycolide) (PLGA) are among the most interesting candidates, since PLGA is a safe and biocompatible polymer that allows both payload protection and target selectivity, if modified with a targeting ligand [10,11]. Protein and hydrophilic drug encapsulation in PLGA NPs is however challenging due to the high hydrophobicity of this material, and several formulations based on double emulsion or modified nanoprecipitation have been studied over the years to improve encapsulation efficiency [12,13,14,15]. We recently developed an enzyme delivery system based on PLGA and exploiting cross-linked enzyme aggregates (CLEAs) tailored to the delivery of therapeutic enzymes involved in lysosomal storage disorders (LSDs) [16]. The use of CLEAs, that are known as excellent biocatalysts in the context of sustainable chemistry [17,18], proved to be successful also in the drug delivery field, resulting in high encapsulation efficiency, activity retention and enzyme activity recovery of in vitro models of LSDs.

Unfortunately most nanomaterials, including the CLEA NP system, are internalized by cells via endocytosis and unavoidably enter the endo-lysosomal pathway, at the end of which the NP and payload are degraded in lysosomes [19,20,21]. While this event is not ominous for the delivery of lysosomal enzymes, whose target is exactly represented by lysosomes, it could severely hamper the efficacy of most molecules of therapeutic interest [22].

Efforts have been made to develop nanocarriers able to overcome the endo-lysosomal pathway and several endosomal escape strategies have been described [23,24,25,26,27]. One of the most promising in terms of clinical potential is represented by the so-called “proton sponge effect” exploited by polymers with buffering capability, typically featuring a large number of primary amines, like poly(ethylene imine) (PEI) [28], poly(amidoamine) [29] and chitosan [30]. When internalized in the acidic lumen of the endosomal vesicle, these polymers become protonated and cause the entrance of protons in the vesicle in order to maintain the endosomal physiologic condition. As a consequence, a parallel influx of chloride ions and water accompanies the influx of protons, leading to an increase of osmotic pressure within the endosome and eventual rupture of the vesicle [29]. PEI has been successfully exploited in cell transfection [31] and in some NP-based delivery systems [32], but to the best of our knowledge no attempts have been made to make PLGA NPs able to carry therapeutic proteins over the endosomal barrier.

In this work, we developed a protein delivery system based on PLGA NPs able to evade the endo-lysosomal pathway and deliver a protein cargo to the cytosol. To do this, we implemented an enzyme delivery system based on CLEAs with an endosomal escape agent (PEI) and we investigated the endosomal escape ability and cellular uptake mechanism of this modified system by means of confocal microscopy, colocalization analysis and endocytosis inhibition. Lastly, we delivered a cytosolic enzyme (superoxide dismutase, SOD) to cultured cells and we measured the NP-mediated antioxidant effect in vitro, demonstrating that active enzymes can be delivered at cytoplasm level. 

## 2. Materials and Methods 

### 2.1. Materials

Poly(D,L-lactide-co-glycolide) (PLGA; Resomer RG 503H) was purchased from Sigma-Aldrich (Darmstadt, Germany) and used as received. Poly(ethylene imine) (PEI), branched, MW 25.000 Da was purchased from Sigma-Aldrich (Darmstadt, Germany) and used as 14 mg/mL stock solution in dimethyl sulphoxide (DMSO). Bovine Serum Albumin (BSA) and Superoxide Dismutase from bovine erythrocytes were purchased from Sigma-Aldrich (Dartmstadt, Germany) and used as received. Atto 488 NHS-ester, Atto 550 NHS-ester and Atto 633 amine were purchased from Atto-TEC GmbH (Siegen, Germany) and used as 10 mg/mL stock solution in DMSO. Fluorescently labelled PLGA was obtained as described elsewhere [16]. Lysotrack Red DND-99 and CellMask Green Plasma Membrane Stain were purchased from Thermo Fisher Scientific (Waltham, MA, USA) and used as received. All other chemicals were purchased from Sigma Aldrich (Darmstadt, Germany) unless specified and used as received.

### 2.2. Cross-linked Aggregates (CLAs) and cross-linked enzyme aggregates (CLEAs) Synthesis

200 µL of 5 mg/mL BSA or SOD solution were added dropwise to 600 µL of acetone simultaneously with 10 µL of 25% glutaraldehyde-water solution under stirring at 4 °C. The mixture was stirred overnight at 4 °C, then centrifuged (30 min, 13,200 rpm, 4 °C) and washed twice with 1 mL of acetone. The final BSA CLAs or SOD CLEAs were suspended in 1 mL of 3% Tween80 in acetone and stored at −20 °C until use. Fluorescently labelled BSA CLAs were prepared as described elsewhere [16].

### 2.3. Nanoparticle (NP) Synthesis

200 µL of BSA CLAs or SOD CLEAs were added to 400 µL of 3% Tween80 in acetone containing 10 mg of PLGA. Then, 0.4 mg of PEI dissolved in DMSO was added to the mixture under sonication bath. This mixture was then added dropwise to 1200 µL of 2% PVA aqueous solution at 37 °C under stirring. The NP suspension was then centrifuged (20 min, 13,200 rpm, 4 °C) and washed twice with 500 µL of deionized water. The final NP pellet was suspended in 50 µL of 100 mg/mL D-(+)-trehalose and stored at −20 °C until use. Fluorescently labelled NPs were prepared with the same protocol but with the addition of 0.1 mg of Atto 633-labelled PLGA and using labelled CLAs; anionic CLAs- or CLEAs-loaded NPs were prepared with the same protocol but replacing PEI with the same volume of DMSO. Empty control NPs were prepared replacing CLEAs with the same volume of 3% Tween80 in acetone.

### 2.4. NP Characterization

#### 2.4.1. Dynamic Light Scattering and Zeta Potential

Hydrodynamic diameter and surface zeta potential of NPs were determined with a ZetaSizer NanoZS (Malvern Instruments Inc., Malvern, UK) by means of dynamic light scattering (DLS). Samples were diluted 1:1000 in deionized water and transferred into polypropylene cuvettes for hydrodynamic diameter measurement or into electrophoretic cells for zeta potential measurement. Mean hydrodynamic diameter was calculated from the autocorrelation function of the intensity of scattered light from NPs by DTS Nano software (version 1.41, Malvern Instruments Inc., Malvern, UK). Electrophoretic mobility was measured and converted into zeta potential by DTS Nano Software.

#### 2.4.2. Protein Quantitation 

The amount of BSA or SOD in NP suspensions was determined via ninhydrine assay [33]. Samples were prepared as follows: 5 uL of NPs or 50 µL of CLAs/CLEAs were digested in 200 µL of 6 M HCl overnight at 95 °C then vacuum dried. The residue was suspended in 50 µL of deionized water, then 10 µL of this solution were added to 110 µL of ninhydrine reagent (20 mg/mL ninhydrine, 2 mg/mL stannous chloride in 75% ethylene glycol in 4N acetate buffer, pH 5.5). The mixture was incubated for 20 min at 95 °C, then samples were plated in a 96-well multiplate and absorbance was measured at 560 nm with a microplate reader (Promega GloMax discover Multimode microplate reader). 

Protein concentration was determined by interpolation from a calibration curve prepared with the BSA or SOD subject to the same digestion and reaction procedures.

### 2.5. Cell Culture

Mouse embryonic fibroblast cells (NIH-3T3) were purchased from the American Type Culture Collection (ATCC, Manassas, VA, USA). Cells were maintained in Dulbecco’s modified Eagle medium (DMEM) from Invitrogen (Carlsbad, CA, USA). Growth medium was supplemented with 10% Fetal Calf Serum (FCS), 4 mM L-glutamine, 1 mM sodium pyruvate, 100 U/mL penicillin, 100 mg/mL streptomycin (Invitrogen). Cells were maintained at 37 °C in a 5% CO_2_ atmosphere. 

### 2.6. Endosomal Escape of NPs

#### 2.6.1. Intracellular Localization of NPs 

Cells were seeded 24 h before experiments onto a glass-bottom Petri dish (WillCo-dish GWst-3522) to reach 80–90% confluence. Incubation with BSA PEI NPs or BSA NPs was performed for 2 h at 37 °C, 5% CO_2_ in DMEM with 10% FCS at final NP concentration of 0.63 or 3 mg/mL, respectively. For immediate imaging after incubation with NPs, cells were washed twice with Phosphate-buffered Saline (PBS) and incubated with 0.1 µM Lysotracker Red DND-99. After 15 min, cells were washed with PBS and medium was replaced with fresh DMEM supplemented with 10% FCS and cells were imaged. For cells imaged 24 hours after incubation with NPs, cells were washed twice with PBS and incubated with fresh medium for 24 h, then stained with Lysotrack and imaged.

#### 2.6.2. Calcein Leakage Assay

Cells were seeded 24 h before experiments onto a glass-bottom Petri dish (WillCo-dish GWst-3522) to reach 80–90% confluence. Incubation with BSA PEI NPs or BSA NPs was performed for 2 h at 37 °C, 5% CO_2_ in DMEM with 10% FCS at final NP concentration of 0.63 or 3 mg/mL, respectively. 30 min before imaging, 1 µL of 50 mg/mL calcein in 1 M NaOH was added to the medium. Then, cells were washed twice with PBS and medium was replaced with fresh DMEM supplemented with 10% FCS and cells were imaged immediately.

#### 2.6.3. Endocytosis Inhibition

Cells were seeded 24 h before experiments onto a glass-bottom Petri dish (WillCo-dish GWst-3522) to reach 80–90% confluence. Cells were placed at 4 °C 15 min before the experiment. Then, incubation with BSA PEI NPs was performed for 45 min at 37 °C, 5% CO_2_ in DMEM with 10% FCS and 160 nM Atto-550 labelled Transferrin and at final NP concentration of 0.63 mg/mL. Then, cells were washed with PBS and fixed with 4% paraformaldehyde before imaging. 

#### 2.6.4. Confocal Imaging

Samples were imaged with a Leica TCS SP5 SMD inverted confocal microscope (Leica Microsystems AG, Vetzlar, Germany) interfaced with Ar, DPSS and HeNe lasers for excitation at 488, 560 and 633 nm, respectively. Live cells were mounted in a thermostated chamber at 37 °C (Leica Microsystems, Vetzlar, Germany) and viewed with a 40 × 1.5 NA oil immersion objective (Leica Microsystems, Vetzlar, Germany) with pinhole aperture set at 1.0 Airy. All images were analyzed using FiJi software (version 2.0.0) and colocalization was determined with JACoP plugin.

### 2.7. SOD Enzymatic Activity

For SOD enzymatic activity assay, the following reagents were prepared shortly before the assay:

(a) Assay buffer (10 mM sodium carbonate, 20 mM EDTA).
(b) 740 μg/mL Nitro Blue Tetrazolium chloride (NBT), 300 μg/mL xanthine in assay buffer (for NP assay) or DMEM (for in-cell assay).
(c) 28 μg/mL Xanthine Oxidase from milk (XOD) in assay buffer (for NP assay) or DMEM (for in-cell assay).

NPs were diluted 1:100 before the assay, then 25 µL of sample were plated in a 96-well multiplate and 50 uL of reagent (b) were added; then, 25 µL of reagent (c) were added and samples were incubated for 30 min at room temperature. Following this, absorbance was measured at 560 nm. 

For SOD enzymatic activity assay in vitro, cells were seeded in a 96-well multiplate 24 h before experiments (10000 cells/well). Cells were then incubated with increasing doses of SOD-loaded NPs in DMEM supplemented with 10% FCS at 37 °C and 5% CO_2_ atmosphere for 6 h. After this time point, cells were washed twice with PBS and incubated with 25 uL of FCS-free DMEM, 50 µL of reagent (b) and 25 µL of reagent (a) for 1 h. Following this, absorbance was measured at 560 nm.

## 3. Results and Discussion

### 3.1. NP synthesis and Characterization

**Figure 1:**
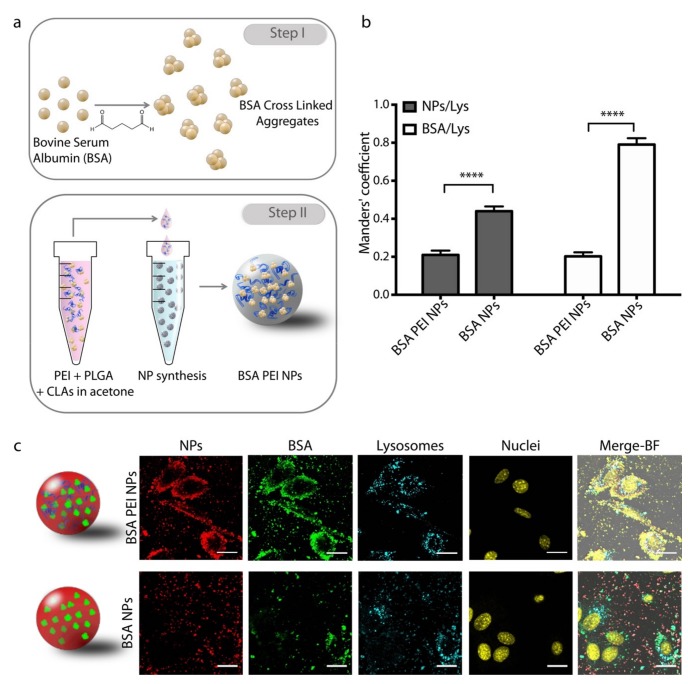
(**a**) Schematic representation of bovine serum albumin (BSA) poly(ethylene imine)(PEI) nanoparticles (NP) synthesis. (**b**) Manders’ coefficient of NP/Lysosomes and BSA/Lysosomes overlap in NIH-3T3 cells. Error bars represent the Standard Error of the Mean, *n* = 10. (**c**) Representative confocal images of NIH-3T3 cells incubated with BSA PEI NPs and BSA NPs imaged 2 h upon treatment. Scale bars: 20 µm.

We modified the previously developed CLEA NPs with an endosomal escape agent in order to achieve the delivery of the protein cargo to the cytosolic compartment. The NP synthesis process was based on our previously described process [16] with the addition of PEI in the PLGA organic phase. To test the effectiveness of our model we first employed Bovine Serum Albumin (BSA) as a protein model and encapsulated into PLGA NPs in form of Cross Linked Aggregates (CLAs). Briefly, BSA was first precipitated in acetone in presence of glutaraldehyde, leading to Schiff base formation and crosslinking of the protein molecules. The resulting BSA CLAs were added to PLGA in acetone, then PEI dissolved in DMSO was added under stirring. This mixture was added dropwise to an aqueous solution of poly (vinyl alcohol) (PVA) under stirring, leading to NP formation (Figure 1a). This way, PLGA precipitates as it enters in contact with the water medium and entraps both BSA CLAs and PEI in form of NPs, that can be collected by centrifugation. We also prepared BSA NPs devoid of PEI as a control.

The obtained NPs were first characterized and compared to control empty NPs, both with and without PEI. All NPs produced showed hydrodynamic diameter ranging from 230 to 280 nm (Table 1), which is suitable for potential in vivo applications [34,35,36], and indicates that the presence of PEI and BSA does not affect the outcome of nanoprecipitation in terms of size. Conversely, PEI strongly affects the surface charge of NPs and leads to cationic NPs, as expected. Indeed, BSA PEI NPs and the respective empty PEI NPs show positive surface Zeta Potential around +40 mV, whereas the same formulation without PEI (BSA NPs and empty NPs) yields negatively charged NPs, with a Zeta Potential around −20 mV which is in line with previously reported results.

The encapsulation efficiency of BSA PEI NPs was determined via ninhydrin assay after protein digestion and compared with BSA NPs.

The presence of PEI does not affect the encapsulation capability of PLGA, leading to similar encapsulation efficiencies for both formulations (45 ± 1% and 47 ± 3%, respectively). This suggests that PEI does not have a specific role in the encapsulation of the payload and that proteins made hydrophobic by CLAs formation are easily embedded by the PLGA matrix itself. 

Another important aspect to be addressed before further experiments is the release profile of NPs [37]. We found that the payload is released with a profile that is typical of PLGA NPs, characterized by an initial burst release followed by sustained release [16,38,39] (Figure S1), which is known to be driven by PLGA erosion and acid-catalyzed hydrolysis at neutral and acidic pH, respectively [40,41].

### 3.2. Intracellular Localization of NPs

Next, we evaluated the intracellular localization of cationic BSA PEI NPs compared to the respective anionic NPs. Prior to this experiment, we investigated the cytotoxicity of PEI NPs, since cationic NPs are frequently reported to be more cytotoxic than anionic NPs [42,43], but no cytotoxic effect was observed at the tested doses (Figure S2). In addition, we assessed the stability of both cationic and anionic NPs in cellular medium and we verified that they do not aggregate nor increase significantly in size (Figure S3).

To evaluate the intracellular localization of NPs, we prepared fluorescently labelled NPs where BSA CLAs and PLGA were tagged with Atto-488 and Atto-633, respectively. NPs were incubated with NIH-3T3 cells for 2 h, then lysosomes and nuclei were stained with specific markers and cells were imaged with a confocal microscope. Images show that BSA PEI NPs are extensively internalized by cells and that NPs are colocalized with BSA, indicating that the protein is not released from the NPs within 2 h (Figure 1c). Both NPs and BSA are distributed in the space between the cell membrane and the perinuclear region, while lysosomes are confined in the latter area. Indeed, BSA PEI NPs are only partially colocalized with lysosomes, as confirmed by the Manders’ coefficient for NP/Lysosome and BSA/lysosome overlaps resulting from the colocalization analysis (Figure 1b). On the contrary, control anionic BSA NPs show a lower degree of internalization and the fraction of NPs that are taken up by cells within 2 h is highly colocalized with lysosomes. Given the intrinsic limitations in the z-resolution of the experimental setup, the distinction between membrane adhesion and internalization would not be trivial, however the colocalization with intracellular organs like lysosomes confirms that at least a significant fraction of NPs is internalized by cells.

Moreover, cells observed 24 h upon administration show that cationic NPs are retained inside cells and spread throughout the whole cell except from the nucleus, including the perinuclear region where lysosomes are located (Figure S4). This inevitably leads to an increase of the Manders’ coefficient for NPs/lysosomes and BSA/lysosomes overlap in this sample (Figure S5). We also observed that at this time point, anionic NPs are internalized by cells and extensively colocalized with lysosomes, as confirmed by the Manders’ coefficient for NPs/lysosomes overlap (above 0.8). However, BSA delivered by anionic NPs displays a lower degree of colocalization with lysosomes compared to the same sample observed 2 h upon incubation. This suggests that BSA is burst released from anionic NPs, a phenomenon which is often observed in non-modified PLGA NPs [44,45], and trafficked within the cells independently from NPs in a shorter time. In fact, BSA is known to be internalized by cells and to follow the endo-lysosomal pathway, at the end of which it is subject to proteolytic degradation [46,47].

### 3.3. Calcein Leakage Assay

**Figure 2:**
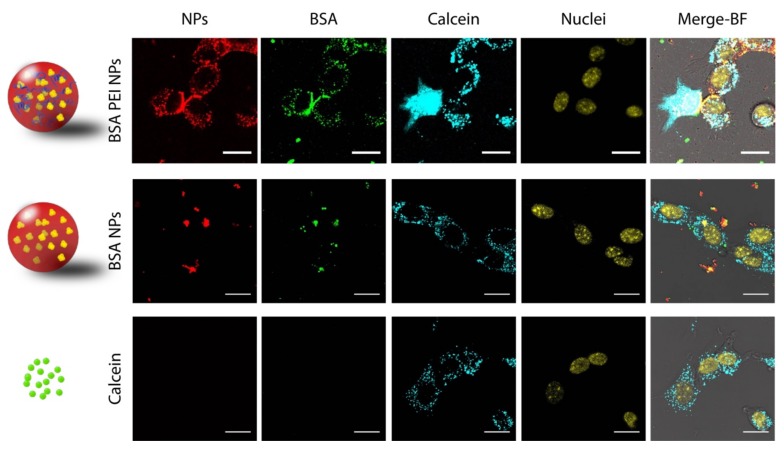
Representative confocal images of NIH-3T3 cells treated with BSA PEI NPs, BSA NPs or Calcein. Scale bars: 20 µm.

To further demonstrate that a fraction of BSA PEI NPs are able to overcome the endosomal barrier, we exploited an assay based on calcein delivery that is commonly used to demonstrate the ability of nanomaterials to disrupt endosomal vesicles [25,48]. When incubated with cells, calcein is taken up via endocytosis and sequestered into vesicles [49]. When vesicles are disrupted and calcein is released in the cytosolic compartment, green fluorescence due to this molecule is observed all over the cell area. Therefore, we incubated either cationic BSA PEI NPs or anionic BSA NPs with NIH-3T3 cells and added calcein to the incubation medium, then we monitored calcein distribution within the cells over the time. To do this, we employed fluorescently labelled NPs that incorporate Atto-633-tagged PLGA and Atto-550-labelled BSA CLAs. Control experiments were also carried out incubating cells with calcein alone. As expected, incubation with calcein alone leads to a punctate fluorescence and no signal is observed in the cytosol and nucleus (Figure 2). The same result is achieved when cells are incubated with anionic BSA NPs. On the contrary, upon treatment with BSA PEI NPs, a fraction of cells in the sample shows intense diffused fluorescence all over the cell in the calcein channel, indicating that the small molecule is released into the cytoplasm. Precisely, we found that around 15% of cells of the analyzed sample reveals diffused fluorescence following incubation with cationic NPs, while almost no cells display diffused fluorescence after treatment with anionic NPs or calcein alone (Figure S6). Taken together, these results confirm what was already evidenced by NP-lysosomes colocalization, pointing out that a fraction of cationic NPs overcomes the endosomal barrier and is therefore not overlapped with lysosomes, while being able to cause the leakage of a small fluorescent molecule and its subsequent release into the cytoplasm.

### 3.4. Internalization Kinetics and Mechanism Study

**Figure 3:**
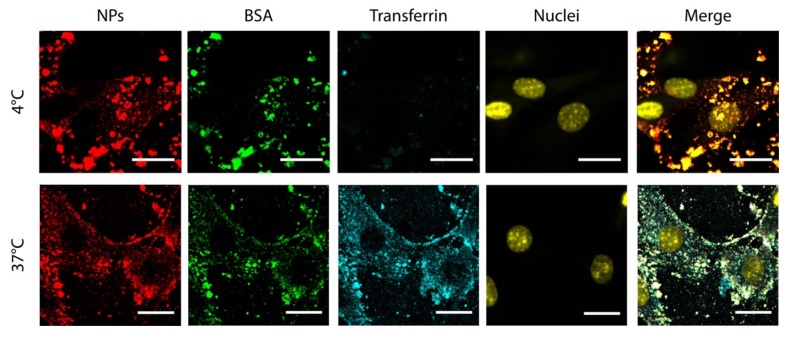
Representative confocal images of BSA PEI NPs internalization in cells at 4 and 37 °C. Scale bars: 20 µm.

At this point, we started to investigate whether cationic NPs are taken up by cells with a different internalization mechanism compared to anionic NPs. Typically, anionic PLGA-based NPs are internalized by endocytosis, a process that requires a certain time to occur (from minutes to hours of incubation) [50,51]. However, cationic NPs employed in this study show a different distribution throughout the cell and are extensively internalized in 2 h, differently from anionic NPs. Thus, it is reasonable to hypothesize that they can enter the cells with a different mechanism. In particular, cationic NPs could enter by direct translocation through the cell membrane exploiting their positive surface charge, as demonstrated for other kinds of cationic NPs [52], completely avoiding the endo-lysosomal pathway, since they would directly enter into the cytoplasm. To understand if the internalization mechanism relies on endocytosis, we treated cells with cationic NPs and blocked the endocytosis process keeping the samples at low temperature during incubation (4 °C) [53,54]. Fluorescently labelled transferrin was added to the incubation medium as a control to ensure that endocytosis is actually inhibited. Control experiments were also performed incubating cells with NPs and transferrin at 37 °C, in a condition that allows internalization by endocytosis. Results show that cationic NPs enter cells even at low temperature, whereas transferrin is internalized only at 37 °C (Figure 3), suggesting that cationic NPs enter cells with a mechanism that does not require ATP like endocytosis [55] but is rather energy-independent, such as direct translocation [56]. Since direct translocation is known to occur within seconds or minutes [57,58], we also performed a time lapse experiment where we started imaging cells immediately after treatment with NPs and in presence of a green fluorescent marker of the plasma membrane. Results show that after 3 minutes of incubation cationic NPs are promptly found either across the membrane or inside the cells, demonstrating that cationic NPs are readily interacting or even being taken up by cells after few minutes (Figure S7). Such a time scale is not compatible with the endocytosis mechanism and supports the hypothesis that cationic BSA PEI NPs could enter cells via direct translocation.

Overall, these data point out that cationic PEI NPs enter cells with a mechanism that completely avoids the endo-lysosomal pathway, and this explains why most NPs are not colocalized with lysosomes. Note however that calcein leakage demonstrate the presence of an alternative pathway based on internalization and endosomal disruption. This fact is supported by the increased NP uptake observed in physiologic conditions and supports the hypothesis that cationic NPs may enter cells also by endocytosis [59], triggering the proton sponge effect that in turn causes endosomal disruption and calcein leakage. Altogether, these evidences and the hypothesis of a mixed internalization mechanism would explain the low percentage of cells that show diffused calcein fluorescence, while supporting the evidence that cationic NPs are able to deliver a protein payload to the cytosolic compartment.

### 3.5. Delivery of a Cytosolic Enzyme

As final proof of the potential of CLAs-loaded PEI NPs as cytosolic protein carriers, we aimed to demonstrate their ability to perform cytosolic delivery of a therapeutic payload. An important group of cytosolic enzymes with therapeutic potential is represented by antioxidant enzymes that control reactive oxygen species (ROS) level inside cells. Such enzymes could be exploited as therapeutics to limit ROS-induced damage, such as cell death, mutations, chromosomal aberrations and carcinogenesis [60]. Among these enzymes, superoxide dismutases (SODs) have been already tested as therapeutics in several delivery systems, such as liposomes [61], mesoporous silica NPs [62], polyketal microparticles [63] and polyion complexes [64]. Indeed, SODs are a group of enzymes that contain metals and can dismute the superoxide anion (O2-) into hydrogen peroxide and molecular oxygen. They can be distinguished according to the metals that they contain, and in particular copper- and zinc-containing SOD, representing 90% of total SOD activity in the cell, is localized in the cytoplasm and in the nucleus [65]. In this work, SOD from bovine erythrocytes was chosen as model to demonstrate the cytosolic delivery of an active enzyme in vitro. 

**Figure 4:**
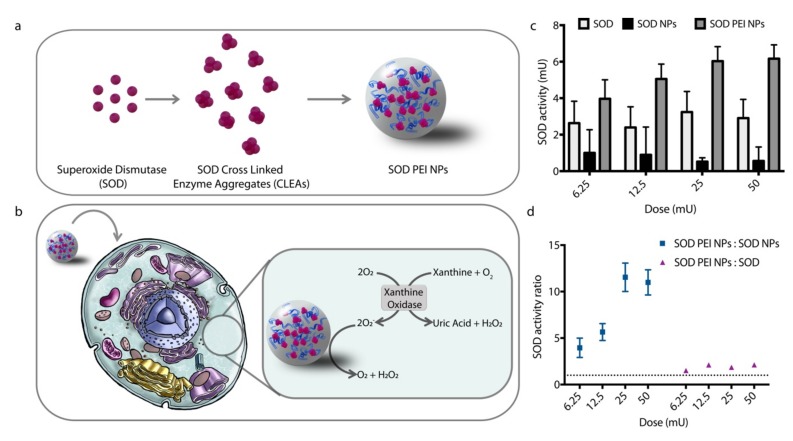
(**a**) Schematic representation of superoxide dismutase (SOD) PEI NP synthesis. (**b**) Schematic illustration of in vitro assay for SOD PEI NPs. (**c**) SOD activity in cell upon treatment with SOD PEI NPs, SOD NPs or SOD. 1 U = 1 nmol non-reduced NBT/min. (**d**) Comparison between SOD activity in cell upon treatment with SOD PEI NPs versus treatment with SOD NPs and SOD. Error bars represent the Standard Error of the Mean, *n* = 3.

First, we prepared SOD cross linked enzyme aggregates (SOD CLEAs) and we encapsulated them into cationic PLGA NPs (Figure 4a). Both the obtained SOD PEI NPs and control SOD NPs show similar encapsulation efficiency and activity yield (around 70% and 30%, respectively, Table 2). Physico-chemical properties (size and zeta potential) were similar to those measured for the previously examined BSA NPs.

We incubated SOD-loaded NPs with NIH-3T3 cells and we measured SOD activity in cell after 6 h. Note that enzyme activity was performed in cells without preliminary lysis, to better evaluate activity arising only from the enzyme localized in cytoplasm. Indeed, preliminary measurements showed that the assay gives a completely negative result when SOD is incubated at pH 4.5 (Figure S8), thus any detected activity in these conditions can only arise from cytoplasmic enzyme and would provide a reliable indication of SOD delivered avoiding or escaping the endo-lysosomal route. We measured enzymatic activity with a well-known assay for SOD based on the reduction of nitro blue tetrazolium (NBT) in presence of xanthine oxidase and xanthine (Figure 4b) [66]. When mixed together, xanthine oxidase converts xanthine into uric acid and hydrogen peroxide and transfers electrons to NBT leading to its reduction to blue formazan, a violet compound that absorbs light at 560 nm. In presence of active SOD, the electron transfer is inhibited and NBT is not converted to blue formazan. Results show that SOD PEI NPs inhibit NBT reduction to a higher extent than free SOD and anionic SOD NPs (Figure 4c). At the highest NP dose the enzymatic activity promoted by cationic NPs is 10 times higher than the enzymatic activity caused by SOD NPs (Figure 4d). This indicates that SOD PEI NPs can perform cytoplasmic delivery of the enzyme. Conversely, anionic SOD NPs efficiently encapsulate active SOD and are taken up by constitutive endocytosis mechanisms. Yet, this approach does not allow increasing SOD enzymatic activity in cells, supporting the evidence that the payload is trafficked into lysosomes and does not reach other compartments. In addition, cells show higher activity upon treatment with SOD PEI NPs also compared to free SOD. It is possible that the tested doses reach saturation of free SOD uptake, leading to the same cell response for all concentrations.

## 4. Conclusions

In summary, we developed a PLGA-based nanostructure for cytoplasmic delivery of enzymes. We validated cell internalization mechanisms and proved that functional enzymes can be easily delivered to the cytoplasm. NP engineering with cationic polymer with proton sponge capability promotes cytoplasmic delivery according to two different and concomitant mechanisms, i.e., direct translocation and endocytosis-endosomal escape. Lastly, as a proof of concept we delivered an active cytosolic enzyme, SOD, in form of CLEAs-loaded PEI NPs to cells and we demonstrated the efficacy of this delivery system also in vitro. NP-mediated delivery, which exploits several different uptake routes, allows more efficient vehiculation of the therapeutic payload. These results open the way to the possibility to perform protein-based therapies which require localization of the active principle in the cytoplasm and, in a more visionary environment, in the nucleus, raising the hope to achieve effective therapies for currently incurable diseases such as amyotrophic lateral sclerosis (ALS) [67] and Canavan disease [68,69]. Indeed, the possibility to functionalize PLGA NPs with ligands targeting the blood brain barrier [16,70] would open the way to the effective brain delivery of cytoplasmic enzymes. Further studies are in progress to validate this delivery strategy for other therapeutically relevant enzymes.

## Figures and Tables

**Table 1:** Hydrodynamic diameter, Zeta potential and Encapsulation Efficiency (EE%) of NPs formulated in this work. SEM = Standard Error of the Mean, *n* = 3.

Formulation	Hydrodynamic Diameter (nm) (SEM)	Zeta Potential (mV) (SEM)	EE% (SEM)
BSA PEI NPs	230 (3)	48.5 (1.0)	45 (1)
BSA NPs	264 (1)	−20.8 (0.6)	47 (3)
PEI NPs	279 (1)	42.7 (0.2)	-
NPs	284 (2)	−25.6 (0.4)	-

**Table 2:** Hydrodynamic diameter, Zeta potential and encapsulation efficiency and activity yield respect to free SOD of SOD-loaded NPs. SEM = Standard Error of the Mean, n = 3.

Formulation	Hydrodynamic Diameter nm (SEM)	Zeta Potential mV (SEM)	Encapsulation Efficiency (%)	Activity Yield (%)
SOD PEI NPs	187 (1)	38 (1)	63 (11)	29 (5)
SOD NPs	321 (9)	−21 (1)	70 (10)	30 (4)

## Supplementary Materials

The following are available online at https://www.mdpi.com/2079-4991/9/4/652/s1, Experimental Procedures: Cell viability assay, Time lapse NP internalization; Figure S1: Payload release from PEI NPs in neutral and acidic buffer; Figure S2: Cell viability of NIH-3T3 fibroblasts upon treatment with increasing doses of cationic PEI NPs; Figure S3: Hydrodynamic diameter of cationic PEI NPs and anionic NPs after 0 and 120 min of incubation at 37 °C in cellular medium. Figure S4: Representative confocal images of NIH-3T3 cells incubated with 488-BSA PEI 633-NPs and 488-BSA 633-NPs imaged 24 h upon treatment; Figure S5: Manders’ coefficient of NP/Lysosomes and BSA/Lysosomes overlap in NIH-3T3 cells.; Figure S6: Percentage of NIH-3T3 cells showing calcein diffused fluorescence upon treatment with BSA PEI NPs, BSA NPs or Calcein; Figure S7: Representative confocal images of NIH-3T3 cells treated with BSA PEI NPs in presence of a specific marker for the plasma membrane after 3 and 45 min of incubation. Figure S8: SOD activity resulting from incubation of free SOD at pH 7.4 or pH 4.5 for 6 h at 37 °C.
