# Supplementary material for: Nanocarriers for Protein Delivery to the Cytosol: Assessing the Endosomal Escape of Poly(Lactide-co-Glycolide)-Poly(Ethylene Imine) Nanoparticles
Source: Nanomaterials (Basel). 2019 Apr 23;9(4):652. doi: 10.3390/nano9040652 (PMC6523739; doi:10.3390/nano9040652)
Supplement: Supplementary file 1 [file nanomaterials-09-00652-s001.pdf]

Supporting Information

Nanocarriers for protein delivery to the cytosol: assessing the endosomal escape of poly(lactide-co-glycolide) - poly(ethylene imine) nanoparticles

Marianna Galliani,*[1,2] Chiara Tremolanti [3,4] and Giovanni Signore* [1,2,5]

[1]Center of Nanotechnology Innovation@NEST, Istituto Italiano di Tecnologia, 56127 Pisa, Italy

[2]NEST, Scuola Normale Superiore, 56127 Pisa, Italy

[3]Department of Pharmacy, University of Pisa, 56126 Pisa, Italy

[4]Istituto di Fisiologia Clinica, National Research Council, 56124 Pisa, Italy

[5]Fondazione Pisana per la Scienza ONLUS, 56121 Pisa, Italy

Supporting Experimental Procedures

Cell viability assay

Cells were seeded in a 96-well microplate 24 hours before the experiments (10.000 cells/well) and maintained at 37°C in a 5% CO2 atmosphere. For cell viability test, cells were incubated with increasing concentrations of PEI NPs (0 mg/ml, 0.63 mg/ml, 1.25 mg/ml, 2.5 mg/ml) or with 20% DMSO in cell medium for 2 hours. Then, medium was removed and cells were washed twice with PBS and incubated with 10% WST-8 reagent in culture medium for 2 hours. Absorbance was then measured at 450 nm with Promega GloMax discover Multimode microplate reader.

Time Lapse NP internalization imaging

Cells were seeded 24 h before experiments onto a glass-bottom Petri dish (WillCo-dish GWst-3522) to reach 80-90% confluence. Cells were treated with 0.63 mg/ml BSA PEI NPs in DMEM with 10% FCS and cell membranes were stained with CellMask Green Plasma Membrane according to the manufacturer's instructions. Cells were mounted in a thermostated chamber at 37°C (Leica Microsystems) and imaging started immediately after treatment. Images of a selected group of cells were acquired every 3 minutes for 45 minutes.

Supporting Figures

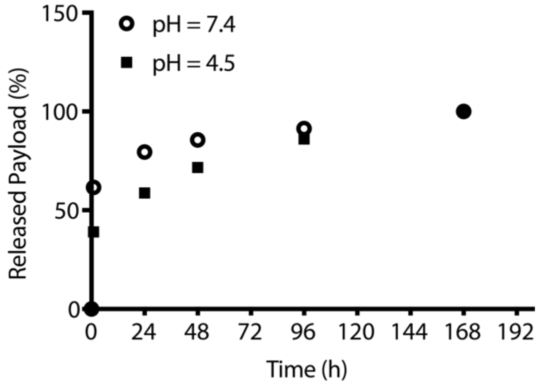

Figure S1. Release profile of PEI NPs in PBS, pH 7.4 or 20 mM acetic acid, 20 mM NaCl buffer (pH 4.5) at 37°C. Results are expressed as % of payload released respect to the total amount of encapsulated cargo.

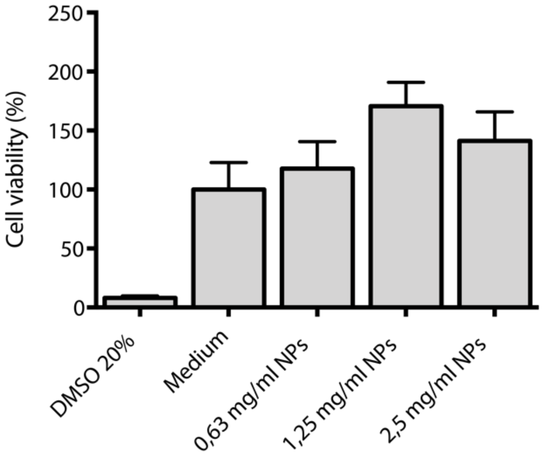

Figure S 2. Cell viability of NIH-3T3 fibroblasts upon treatment with increasing doses of cationic PEI NPs. Error bars represent the standard error of the mean, n = 3.

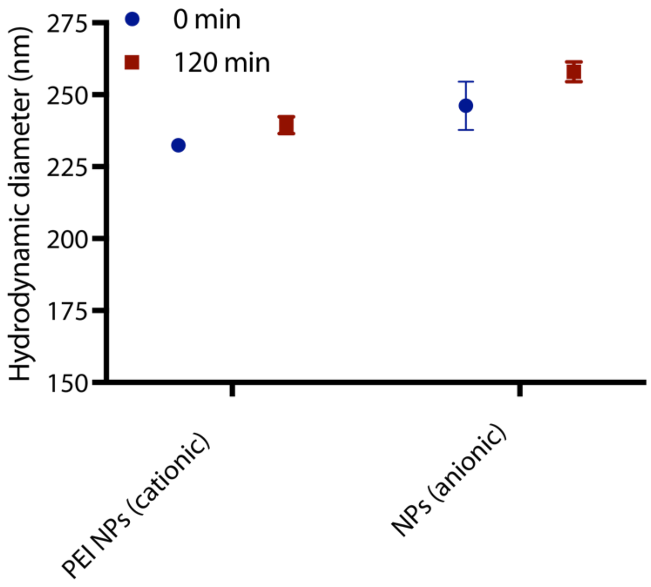

Figure S 3. Hydrodynamic diameter of cationic PEI NPs and anionic NPs after 0 and 120 minutes of incubation at 37°C in DMEM supplemented with 10% Fetal Calf Serum (FCS), 4 mM L-glutamine, 1 mM odium pyruvate, 100 U/ml penicillin, 100 mg/ml streptomycin. Error bars represent the standard error of the mean, n = 3.

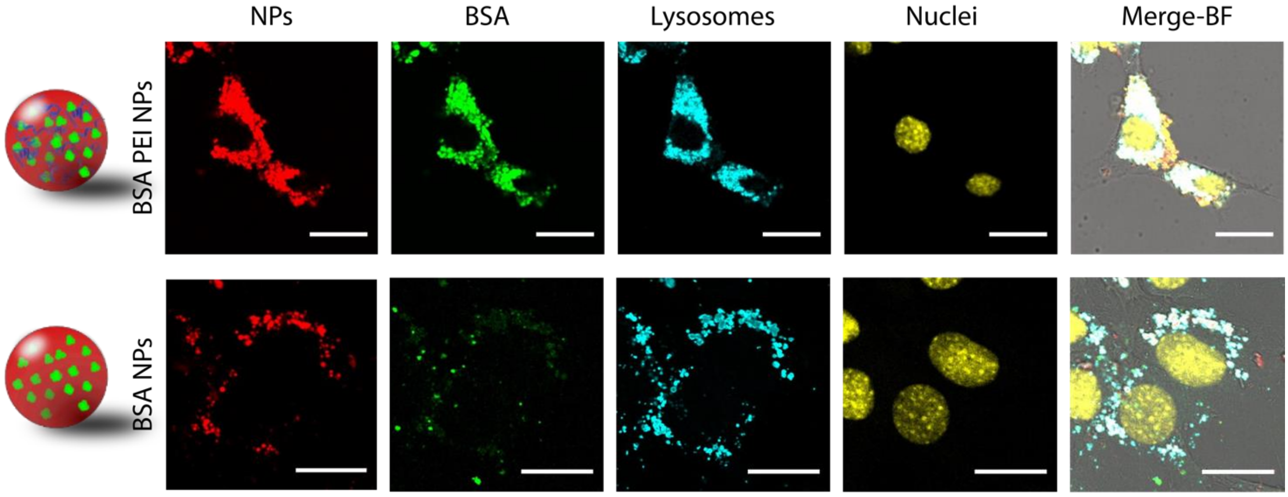

Figure S 4. Representative confocal images of NIH-3T3 cells incubated with 488-BSA PEI 633-NPs and 488-BSA 633-NPs imaged 24 hours upon treatment. Scale bars: 20 uM

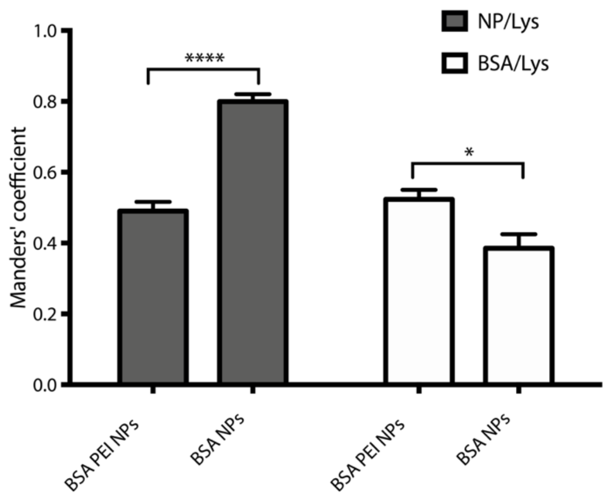

Figure S 5. Manders’ coefficient of NP/Lysosomes and BSA/Lysosomes overlap in NIH-3T3 cells. Error bars represent the Standard Error of the Mean, n = 10.

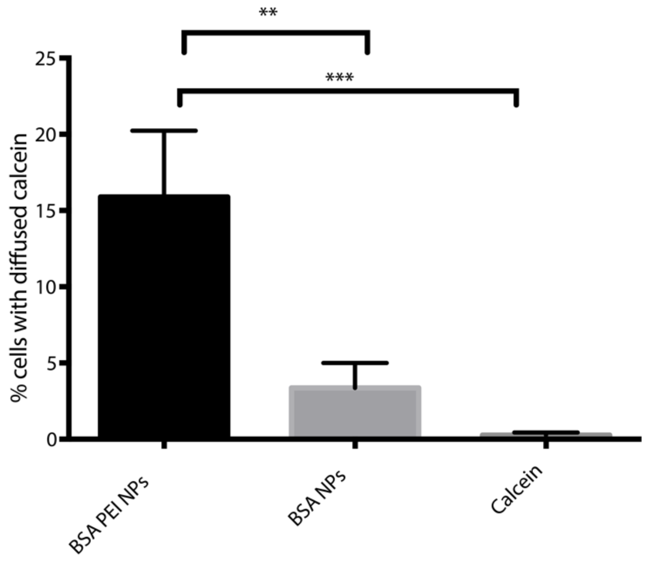

Figure S 6. Percentage of NIH-3T3 cells showing calcein diffused fluorescence upon treatment with BSA PEI NPs, BSA NPs or Calcein.

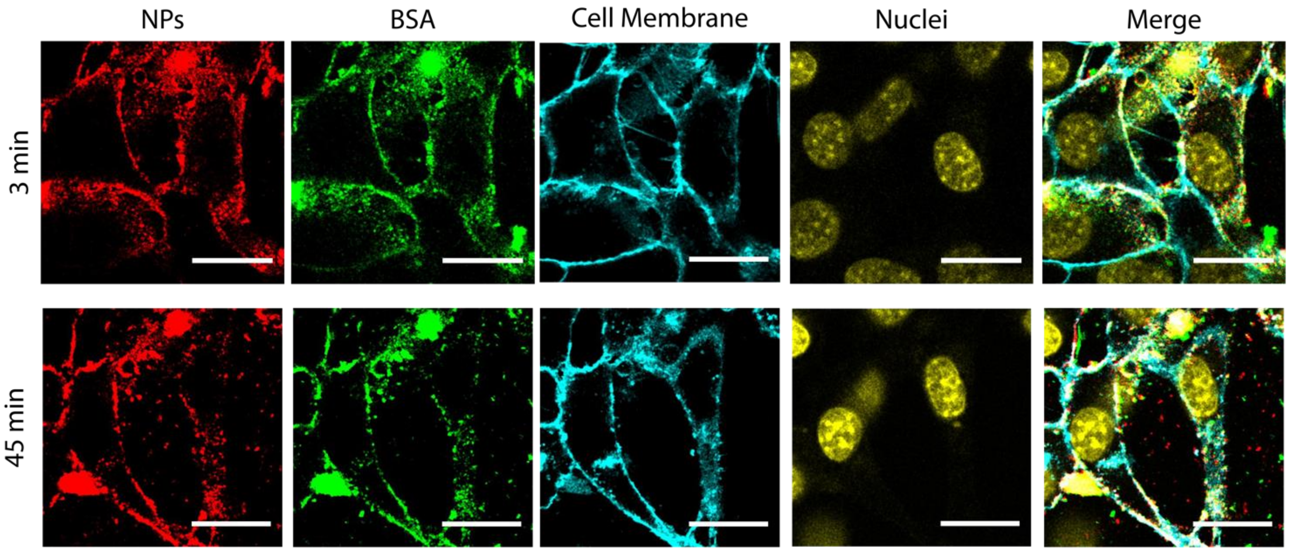

Figure S 7. Representative confocal images of NIH-3T3 cells treated with BSA PEI NPs in presence of a specific marker for the plasma membrane after 3 and 45 minutes of incubation. Scale bars: 20 uM.

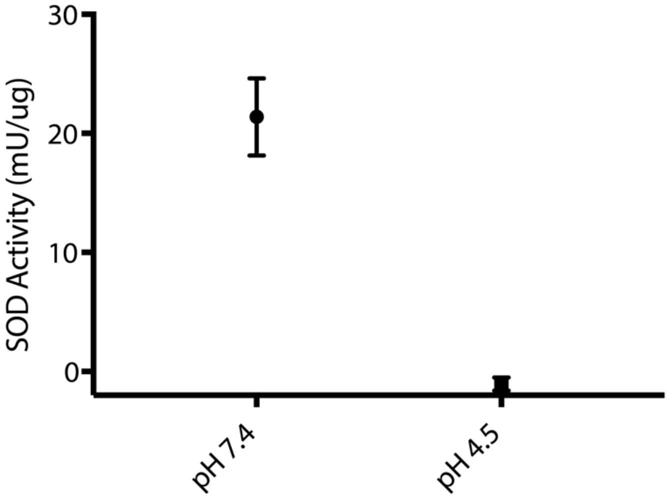

Figure S 8. SOD activity resulting from incubation of free SOD at pH 7.4 or pH 4.5 for 6 hours at 37°C. 1 U = 1 nmol non-reduced NBT/min. Error bars represent the Standard Error of the Mean, n = 3.
